# Exposure of *Allium cepa* Root Cells to Zidovudine or Nevirapine Induces Cytogenotoxic Changes

**DOI:** 10.1371/journal.pone.0090296

**Published:** 2014-03-05

**Authors:** Chika K. Onwuamah, Sabdat O. Ekama, Rosemary A. Audu, Oliver C. Ezechi, Miriam C. Poirier, Peter G C. Odeigah

**Affiliations:** 1 Human Virology Laboratory, Nigerian Institute of Medical Research, Yaba, Lagos, Nigeria; 2 Clinical Sciences Division, Nigerian Institute of Medical Research, Yaba, Lagos, Nigeria; 3 Carcinogen-DNA Interactions Section, LCBG, Center for Cancer Research, National Cancer Institute, NIH, Bethesda, Maryland, United States of America; 4 Department of Cell Biology & Genetics, University of Lagos, Akoka, Lagos, Nigeria; Fudan University, China

## Abstract

Antiretroviral drugs have proved useful in the clinical management of HIV-infected persons, though there are concerns about the effects of exposure to these DNA-reactive drugs. We investigated the potential of the plant model *Allium cepa* root tip assay to demonstrate the cytogenotoxicity of zidovudine and nevirapine and as a replace-reduce-refine programme amenable to resource–poor research settings. Cells mitotic index were determined in squashed root cells from *Allium cepa* bulbs exposed to zidovudine or nevirapine for 48 hr. The concentration of zidovudine and nevirapine inhibiting 50% root growth after 96 hr exposure was 65.0 µM and 92.5 µM respectively. Root length of all antiretroviral-exposed roots after 96 hr exposure was significantly shorter than the unexposed roots while additional root growth during a subsequent 48 hr recovery period in the absence of drug was not significantly different. By ANOVA, there was a significant association between percentage of cells in mitosis and zidovudine dose (*p* = 0.004), but not nevirapine dose (*p* = 0.68). Chromosomal aberrations such as sticky chromosomes, chromatin bridges, multipolar mitoses and binucleated cells were observed in root cells exposed to zidovudine and nevirapine for 48 hr. The most notable chromosomal aberration was drug-related increases in sticky chromosomes. Overall, the study showed inhibition in root length growth, changes in the mitotic index, and the induction of chromosomal aberrations in *Allium* bulbs treated for 96 hr or 48 hr with zidovudine and nevirapine. The study reveals generalized cytogenotoxic damage induced by exposure to zidovudine and nevirapine, and further show that the two compounds differ in their effects on mitosis and the types of chromosomal aberrations induced.

## Introduction

The human immunodeficiency virus (HIV) is the retrovirus that causes the acquired immunodeficiency syndrome (AIDS). Currently there is no known curative therapy and continuous exposure to combinations of several antiretroviral (ARV) drugs is required for clinical management. However, there are concerns regarding the adverse effects of ARV therapy, particularly the perinatal exposures [1], [2], [3], [4]. Clinical reports addressing the effects of perinatal ARV exposures in children born to HIV-positive mothers have been varied. Culnane *et al.*, [5] reported that the biometrics of such exposed children were not statistically different from unexposed children, and Paul *et al.*, [6] and Briand *et al.*, [7] found some adverse effects, which were inconsequential, and reversible over time. However, evidence of persistent left ventricular muscle loss was reported by Lipshultz *et al.*, [8] to occur in some HIV-1-uninfected children born to HIV-1-infected mothers, and to be persistent for up to 2 years of age. The reversibility of these events over time has not been reported on. Furthermore, the glycophorin A (GPA) somatic cell mutation assay, which screens for large-scale DNA damage in red blood cells, showed clear evidence that GPA variants were significantly elevated in mother-child pairs exposed to zidovudine and lamivudine compared to unexposed individuals [9]. The elevated GPA variants persisted through one year of age justifying the need for long-term surveillance [9]. It is important to clarify the nature of any ARV-induced genotoxic effects, and to ascertain if these effects resolve when the drugs are withdrawn.

To investigate these, we thought to use the *Allium* model routinely used to evaluate chemicals and for environmental monitoring. However, we need to first determine the suitability of the *Allium cepa* root tip assay in demonstrating the cytotoxicity of the antiretroviral drugs zidovudine (ZDV) and nevirapine (NVP). Thereafter we sought to determine if any effects on root growth observed resolve when the drugs are withdrawn. The *Allium cepa* model is a plant model, easy to handle and the chromosome condition of plant cells is good, providing a high standard in the unexposed controls. *Allium* has large chromosomes that are easily visualized and lend themselves to studies of drug-induced chromosomal aberrations. For genotoxic studies, the *Allium* test is relatively rapid, easy to perform, highly sensitive, reproducible and provides results comparable to other prokaryotic and eukaryotic test systems [10].

If the cytogenotoxicity of zidovudine and nevirapine are established to be inducible in *Allium cepa*, the large-sized chromosomes of this model could then be used in further studies. Certain earlier reported toxic effects of the drugs were observed and are reported here.

## Materials and Methods

### Study materials

This study was approved by the Institutional Review Board of the Nigerian Institute of Medical Research. Locally available *Allium cepa* (purple variety) bulbs used for this study were purchased from the Mile 12 market in Lagos, Nigeria. Two hundred and four medium-sized *Allium cepa* bulbs (42–53 mm) were used. Generic paediatric syrup formulations (10 mg/ml) of the drugs ZDV and NVP used were obtained from the Pharmacy Unit of the antiretroviral clinic of the Nigerian Institute of Medical Research (NIMR), Lagos.

### Root growth inhibition with ZDV and NVP for 96 hr, and recovery after drug removal for 48 hr

The first experiment involved growing the onions for 96 hours in 6 concentrations of drug, and measuring root growth. The clinical peak plasma ZDV level is about 10 µM, and the doses of ZDV and NVP used were 0, 10, 100, 200, 400, 800 and 1200 µM prepared from the 10 mg/ml syrup. The test drug concentrations were prepared by diluting the syrups with deionized water, where the smallest volume of syrup was 27 µl in 100 ml water, and the largest was 3240 µl in 100 ml water. The volume needed per concentration was prepared once, stored at 2–8°C in aliquots sufficient for daily use and added fresh to the *Allium* bulbs every 24 hours. The drug solutions were changed daily being a semi-static study to ensure drug concentrations were relatively constant. Aliquots were allowed to reach room temperature and mixed thoroughly before use each day. Each *Allium* bulb was grown in a transparent glass bottle containing about 50 ml of the drug concentration. The root growth length per dose was measured, allowing calculation of the half maximal effective concentration (EC_50_) or the dose that inhibited root growth length by 50%. These experiments revealed the toxicity of ZDV and NVP with respect to root growth. Six *Allium* bulbs were used per concentration, but at the time of harvest the *Allium* bulb with the poorest root growth was discarded as per standard protocol [10], [11]. The root bundle length of the five remaining *Allium* bulbs was measured, and the root length in drug-exposed groups was expressed as a percentage of the root length in the negative control. The value for EC_50_ was then obtained from a plot of the growth curve for each drug.

After 96 hr of ARV drug exposure, the drug was removed and the five *Allium* bulbs evaluated per concentration were grown for an additional 48 hours in deionized water. The deionized water was changed every 24 hours. At 48 hours of growth in deionized water, the root length measurements were repeated for all the *Allium* bulbs, to determine if the root growth inhibition in the presence of the test drugs was reversible.

### Chromosomal aberrations after 48 hr of Allium bulb growth in ZDV or NVP

The chromosomal aberration test was performed according to Odeigah *et al.*, [11] using an unexposed group and four doses of each drug, 6.5–65.0 µM ZDV and 9.3–92.5 µM NVP. The earlier determined EC_50_ of each drug was the highest dose used here. Deionized water was used as drug solvent. All *Allium* bulbs were first grown in deionized water for 24 hr before transfer to the drug solutions for another 48 hr (about 2 cell cycles). The test solutions were changed every 24 hr and the *Allium* bulbs were exposed to the drugs for a total period of 48 hours. Twelve *Allium* bulbs were exposed to each drug concentration but the 2 with the poorest growth were excluded for each concentration. The remaining ten *Allium* bulbs were then prepared for microscopy using the conventional aceto-orcein squash technique [11]. Briefly, two to three root tips from one *Allium* bulb were harvested, fixed, and macerated in a solution of 9 parts 45% acetic acid and 1 part 1N HCl for 5 min. Aceto-orcein stain was then dropped on the squashed material. The cover slip was used to press down and spread the squashed materials. The edges of the coverslip were then sealed with nail varnish to reduce fluid evaporation. Thus ten slides per concentration were prepared and coded, and 40–100 cells per slide were examined, scored for mitotic index (percentage dividing cells or cells in mitosis) and for chromosomal aberrations according to Odeigah *et al* [11].

### Statistical evaluation

Statistical comparisons were performed using the replicate values for each group by t-test (MS Excel; two sample unequal variance), where a *p* value less than 0.05 was considered statistically significant. Analysis of variance (ANOVA) was performed to compare unexposed controls with the ZDV and NVP treatment group responses for percentage of cells in mitosis.

## Results

### Comparative genotoxicity and EC_50_ values from the 96 hr root growth inhibition assay

**Figure 1:**
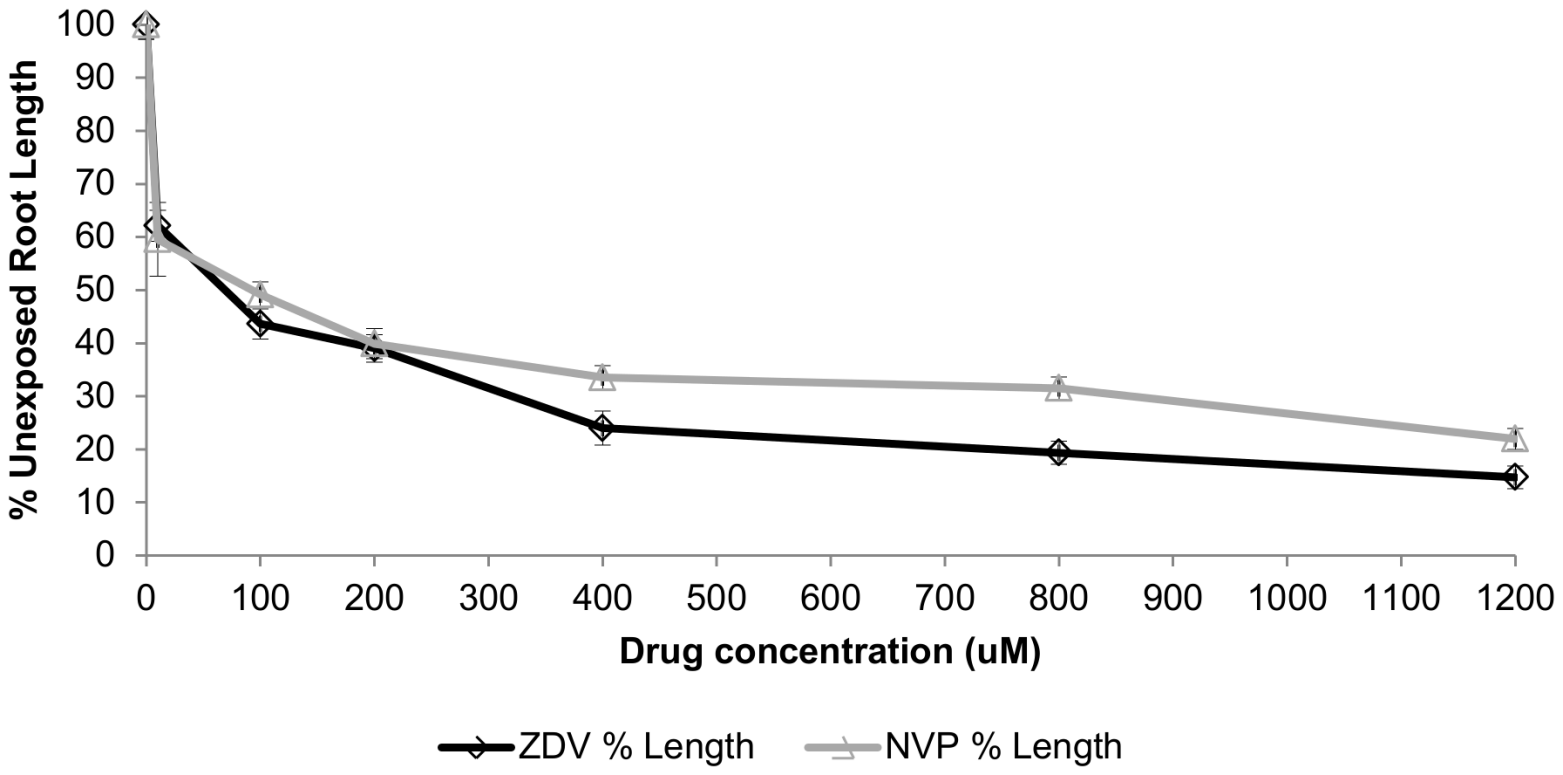
*Allium cepa* root length grown for 96 hr in zidovudine (black line) or nevirapine (grey line). Expressed as percentage of the unexposed control with value 34.6±2.7 mm (mean ± SE; n = 5 bulbs/group). The drug concentration giving half maximal root growth length (EC_50_) for zidovudine (ZDV) was 65.0 µM, and for nevirapine (NVP) was 92.5 µM. At the 400, 800 and 1200 µM doses, roots from the NVP-exposed groups were significantly longer than those from the ZDV-exposed groups.

**Figure 2:**
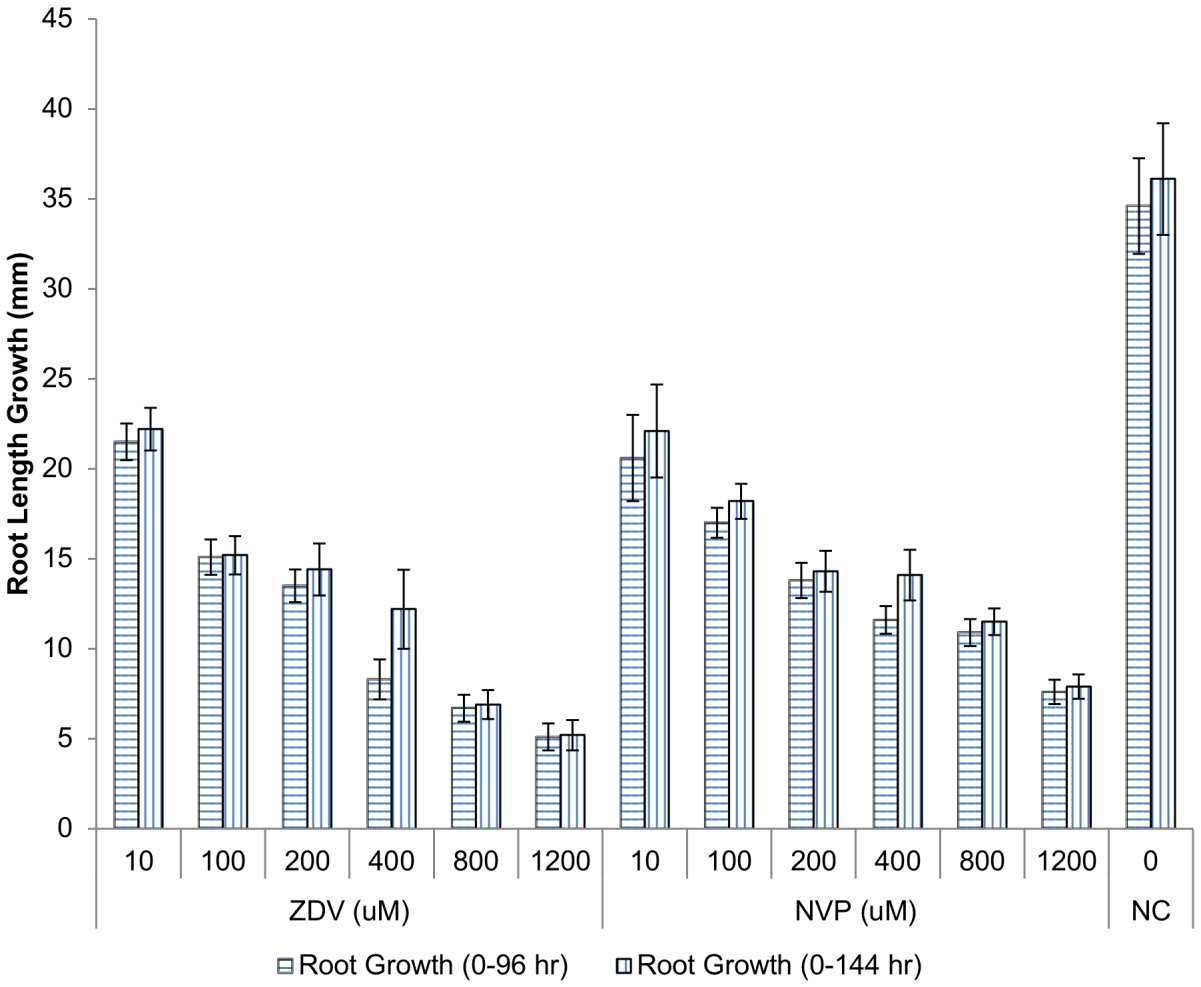
Root length during the 96(horizontal stripes) and 144 hr (vertical stripes) growth periods. For 96(NC  =  negative control), ZDV or NVP. From 96 hr to 144 hr (vertical stripes), all bulbs were grown in water without drug. Root length (mm) shown is mean ± Standard error, with n = 5 bulbs per group.

When average root growth at 96 hours in untreated bulbs was compared to the growth seen in the presence of either ZDV or NVP, all the ARV-exposed bulbs had significantly (*p*≤0.005) shorter root lengths. Dose-related responses were observed for both drugs (Figures 1 and 2). Figure 1 shows the root length for each drug dose, expressed as a percentage of the average unexposed root length. The data indicates that ZDV is more toxic than NVP at the three highest concentrations. This observation is further supported by the EC_50_ values, which were calculated from the data shown in Figure 1, where 65.0 µM ZDV and 92.5 µM NVP were the concentrations giving 50% inhibition of root growth length. Figure 1 also shows that the root growth lengths observed with ZDV- and NVP-treated bulbs were similar at doses lower than 400 µM, but that values with ZDV at 400 µM (*p* = 0.044), 800 µM (*p* = 0.004) and 1200 µM (*p* = 0.039), were all significantly shorter than those seen with NVP.

### Evaluating the reversibility of ARV drug toxicity during 48 hours in the absence of drug

The root growth length (mm) obtained during 96 hr of exposure to ZDV, NVP or no drug, is shown in Figure 2 (bars with horizontal lines). The same bulbs, from 96 to 144 hours, were grown in deionized water without drug, to evaluate the possibility of recovery from ARV-induced toxicity, and the results are shown in Figure 2 (bars with vertical lines). During the 48 hours recovery stage, there was some additional growth (between 0.7–47.0% of their initial length) for all the concentrations. The average cumulative root length (mm) after 144 hr for the different concentrations (10, 100, 200, 400, 800, 1200 µM) were 22.2, 15.2, 14.4, 12.2, 6.9 and 5.2 for ZDV and 22.1, 18.2, 14.3, 14.1, 11.5 and 7.9 for NVP. Compared to the unexposed roots, all test groups were significantly (all p≤0.009) less than the root length recorded for the unexposed roots (36.1±3.1 mm) after 144 hr, with most being less than half of it root length (Figure 2).

Comparing the ZDV and NVP cumulative (144 hr) root growth lengths to each other, only the 800 uM (*p* = 0.003) and 1200 µM (*p* = 0.039) concentrations showed treatment-related differences, and the NVP-exposed bulbs had longer root lengths at both concentrations. At the other drug doses, cumulative root growth lengths (mm) for ZDV and NVP were not significantly different from each other, though all were significantly shorter than the cumulative length of the unexposed roots. At 400 µM of both ZDV and NVP, the growth during hours 96–144 was greater than that seen in the negative controls (Figure 2), but at the other ZDV and NVP doses, growth during the 96–144 hr recovery period showed no consistent pattern.

### Mitotic index patterns observed during the 48 hours of exposure to ZDV or NVP

Cells in various phases of mitosis after 48 hr of exposure to ZDV or NVP were evaluated by light microscopy and the results are shown in Table 1 as percentages of cells in mitosis. The data for mitotic index show mean ± SD for n = 10 bulbs/group. The percentage of cells in mitosis for the unexposed control group (4.1%) was significantly lower than the 7.6% observed in the group exposed to 32.5 µM ZDV (*p* = 0.02). In addition, for the percentage of cells in mitosis in the 46.3 µM NVP group, 2.9%, was significantly lower (*p* = 0.03) than the 4.1% for the unexposed controls. The relationship between percentage of cells in mitosis and drug concentration was non-linear and not dose-dependent for both the ZDV and the NVP groups. However, ANOVA analysis of the relationship between drug dose and percentage of cells in mitosis, showed a significant correlation for ZDV (*p* = 0.004), and none for NVP (*p* = 0.68).

**Table 1:** Impact of ZDV and NVP on the mitotic index of *Allium cepa* root cells^a^ exposed for 48 hr.

Chemical Exposure (μM)	EC_50_ (%)^b^	Cells evaluated (n)	Percentage of cells in Mitosis
None	-	1000	4.1±2.3
6.5 ZDV	10	736	6.5±3.3
16.3 ZDV	25	744	5.5±2.6
32.5 ZDV	50	747	7.6±3.4^c^
65.0 ZDV	100	690	2.7±2.1
9.3 NVP	10	503	5.3±5.4
23.1 NVP	25	361	4.1±4.8
46.3 NVP	50	429	2.9±5.2^c^
92.5 NVP	100	720	5.0±3.5

a Each experimental group contained 10 bulbs and 2–3 root tips from each bulb were squashed together on one slide. On each slide 40–100 cells were examined. Values shown are means ± standard deviation of the mean.

b EC_50_ is the concentration of drug that inhibits *Allium* root growth length by 50%; 10% of EC_50_ is 5% of total root growth length.

c For the percentage of cells in mitosis: for unexposed vs.32.5 µM ZDV, *p* = 0.02; for ZDV 50% of EC_50_ vs. NVP 50% of EC_50_, *p* = 0.03. All other associations were statistically non-significant.

### Chromosomal aberrations observed during 48 hours of ZDV or NVP exposure

**Figure 3:**
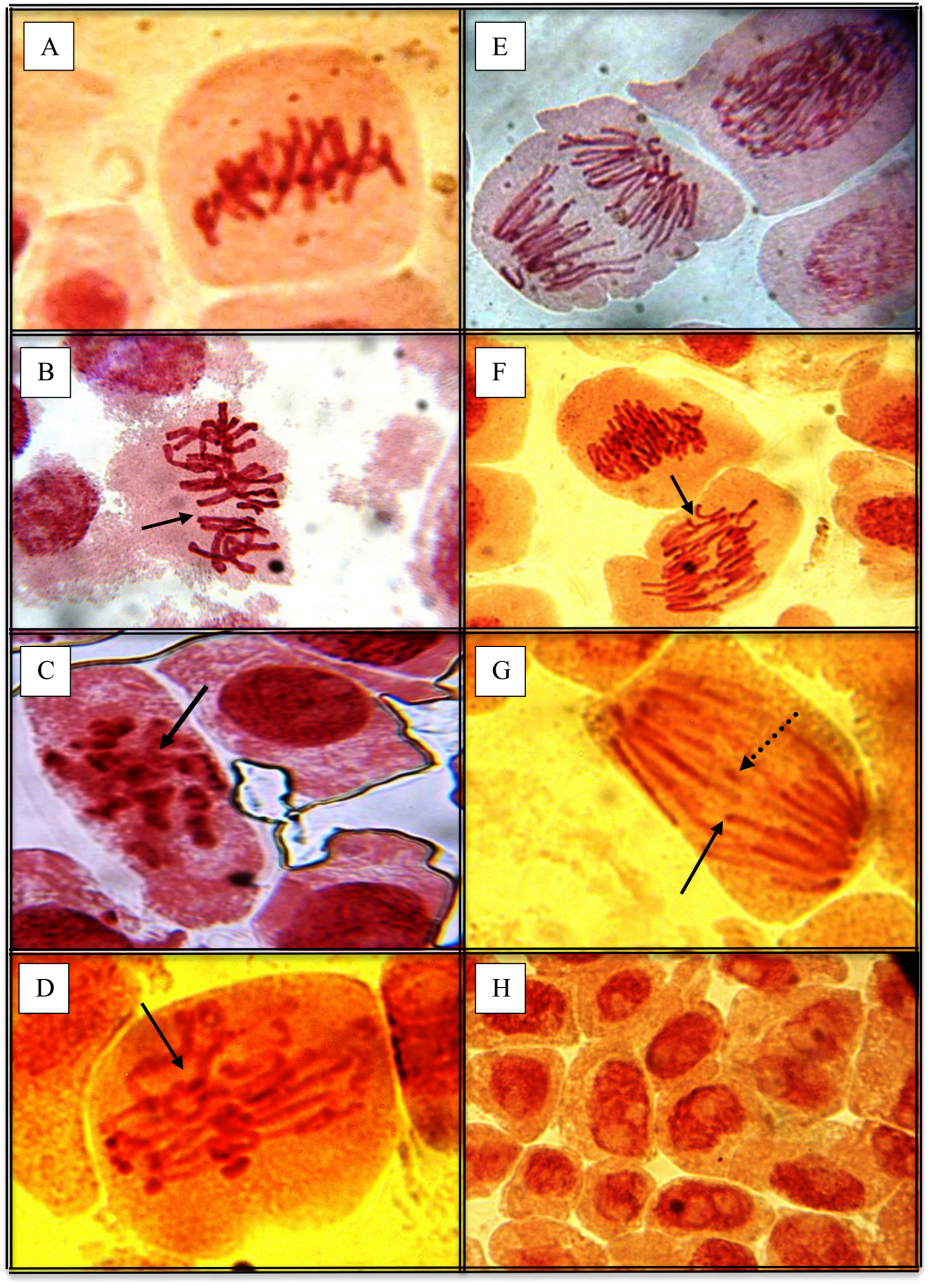
Micrographs of chromosomal aberrations seen in the treatment groups. A – Normal metaphase from the negative control group. *B – Vagrant metaphase form with misaligned chromosomes in cell from 32.5 µM ZDV. *C – C-Metaphase chromosomes seen only in cells exposed to 32.5 µM ZDV. D – Cell exposed to 65 µM ZDV showing sticky chromosomes. E – Normal anaphase (early) from the negative control group. F – Mitotic figure from cell exposed to 6.5 µM ZDV showing anaphase bridges (arrowed). G – Mitotic figure showing cell exposed to 6.5 µM ZDV; arrows indicate uneven breaks (black) and chromosomal fragment (patterned). H – Interphase cells exposed to 6.5 µM ZDV indicating interphase arrest. *Challenges with instruments delayed taking micrographs till some slides started drying.

The chromosomal aberrations found in *Allium* bulb root cells after 48 hrs of exposure to no drug (1000 cells/group), ZDV (690–747 cells/group), or NVP (361–720 cells/group), include sticky chromosomes, chromatin bridges, vagrant forms, binucleated cells, multipolar mitosis and chromosomal fragments (as shown in Table 2). Many of these types of aberrations can be seen in Figure 3, where all the photos show cells exposed to ZDV. None of these aberrations was seen in unexposed cells. The highest frequency of total chromosomal aberrations, in the ZDV-exposed groups, was seen at 65 µM ZDV (Table 2), a dose shown in Table 1 to have substantial cell cycle abnormalities. In this group, sticky chromosomes (57.9%) and binucleated cells (5.3%) were the only aberrations, suggesting that sticky chromosomes comprise a majority of aberrations formed under conditions of toxicity. Sticky chromosomes (Figure 3A) refer to the tendency of chromosome arms or entire chromosomes to stick together, increasing the chance for rearrangement events. A high percentage of sticky chromosomes, 61.9% and 56.8%, were also seen in cells exposed to the two highest doses of NVP, 46.3 µM and 92.5 µM, respectively. In general the variety of aberrations was greatest in the groups exposed to 6.5–32.5 µM ZDV, doses which showed anaphase bridges (Figure 3B), vagrant metaphases (Figure 3C), multipolar spindles (Figure 3D), chromosomal fragments (Figure 3E) and binucleated cells (Figure 3F). In contrast, all the groups exposed to NVP had a predominance of sticky chromosomes, with only one or two other types of aberration.

**Table 2:** Percentage of *Allium* cells with various chromosomal aberrations after exposure for 48 hr to ZDV or NVP.

Chemical Exposure (μM)	Cells evaluated (n)	Sticky chromosomes^a^ (%)	Chromatin bridges^b^ (%)	Vagrant forms^c^ (%)	Binucleate (%)^d^	Multipolar mitosis^e^ (%)	Chromosomal Fragments^f^ (%)	Total Aberration (%)
None	1000	0	0	0	0	0	0	0
6.5 ZDV	736	25.0	12.5	6.3	4.2	-	6.3	56.3
16.3 ZDV	744	23.3	7.0	16.3	2.3	2.3	-	55.8
32.5 ZDV	747	24.6	8.8	12.3	3.5	1.8	3.5	56.1
65.0 ZDV	690	57.9	-	-	5.3	-	-	63.2
9.3 NVP	503	36.0	-	20.0	-	-	-	56.0
23.1 NVP	361	53.3	-	6.7	-	-	-	60.0
46.3 NVP	429	61.9	-	-	4.8	-	-	66.7
92.5 NVP	720	56.8	-	2.7	5.4	-	-	64.9

a *Sticky chromosomes* refer to the tendency of chromosome arms or entire chromosomes to stick together.

b *Chromatin bridges* occur in mitosis when the telomeres of sister chromatids fuse together and fail to completely segregate into their respective daughter cells. ^c^ *Vagrant forms* refer to chromosomal formations different from the normal formation during mitosis.

d *Binucleated cells* have more than one nucleus. ^e^ *Multipolar mitosis* occurs when the chromosomal material is pulled to more than two poles, resulting in the formation of a corresponding number of nuclei. ^f^ *Chromosomal fragments* refer to fragments of a chromosome that may be lacking a centromere and so is often lost when the cell divide.

## Discussion

In this study we have demonstrated the genotoxicity of ZDV and NVP for *Allium* root growth, for changes in mitotic index, and for chromosomal aberrations. Taken together all of these events show clear differences between the genotoxic effects of ZDV and NVP, with ZDV being more inhibitory to root growth at high concentrations, causing more alterations in mitosis, and inducing more chromosomal aberrations, than NVP. The study demonstrates the potential of the *Allium cepa* model for evaluating the genotoxic effects of antiretroviral NRTI drug exposures. These studies suggest that the model could be useful for screening antiretroviral drugs that do not require metabolism, and evaluating genotoxic effects in situations where it is not possible to employ an animal model or where cell culture studies are inadequate to reveal the variety of events in a whole organism. This model is particularly useful for cytogenetic work because of the large and easily-visible chromosomes.

The *Allium* root growth inhibition assay, carried out for 96 hr, showed that ZDV is more toxic than NVP, especially at the higher concentrations of 400–1200 µM, where root lengths in onions exposed to ZDV were significantly shorter than with NVP. The lower EC_50_ value obtained for ZDV in this study further suggests that it is more toxic than NVP. Our results are intriguing and suggestive of DNA replication-based perturbation caused by these two HIV reverse transcriptase inhibitors. Therefore the effects observed here may relate directly to a replication DNA polymerase in *Allium cepa*. More work with the specific DNA polymerases of *Allium cepa* and their inhibition in vitro by ZDV and NVP is required.

For both drugs, under 400 µM there was additional growth during the 96–144 hr post-drug recovery period. However at the 800 and 1200 µM doses for both drugs, there was minimal additional growth during the recovery period. This suggests that the genotoxic effects at those higher concentrations may not be reversible. The 96–144 hr recovery data further suggests that the drug-induced root growth inhibition could not be fully reversed, as most groups had cumulative root growth less than half of that exhibited by the negative control. However, during the 48 hr recovery period the negative control also had reduced root growth, suggesting that growth had slowed down naturally.

In addition to root growth length, we examined mitotic index parameters in bulbs exposed for 48 hr to a range of ZDV and NVP concentrations. Most groups showed values for % of cells in mitosis that were similar to the unexposed controls, with the exceptions of 65.0 µM ZDV and 46.3 µM NVP, which gave significantly different values for % of cells in mitosis. In these studies we found many cells synchronized in interphase across the various drug concentrations, suggesting an interphase arrest. Furthermore, the NVP-treated *Allium* root cells had few cells in metaphase and anaphase, as most cells were in telophase. It appears that NVP-treated cells escaping from the interphase arrest had their metaphase and anaphase shortened, accumulating cells again in telophase. This alteration in the mitotic phase distribution agrees with the induction of premature senescence. ZDV had been earlier reported to arrest HeLa and other cells in S-phase [12]. NVP has also been variously reported to induce premature senescence and accumulate HeLa cells in G1 phase [13], [14]. Thus the findings of this study agree with the reported effects of ZDV and NVP on the S-phase and the G1 phase of cell division in HeLa cells [12], [13], [14].

Chromosomal aberrations were also observed in these studies. Chromosomal stickiness was the major chromosomal anomaly recorded, and occurred in a dose-responsive pattern. During cell division, sticky chromosomes could produce aneuploid or polyploid cells. Stickiness, combined with aberrations such as chromosome bridges and vagrant chromosome formations, may increase the potential for micro- or macro-nucleus formation. Formation of micronuclei containing whole chromosomes produces aneuploidy. Chromatin bridges occur in mitosis when the telomeres of sister chromatids fuse together and fail to completely segregate into their respective daughter cells. Chromosome fragments (micronuclei) that lack a centromere are often lost when the cell divides. Vagrant forms may result from weak spindle formations. Multipolar mitoses occur when centrosomal amplification results in more than two spindles, making it almost impossible for cells to acquire proper distribution of chromosomes. In this study, the chromosome stickiness and bridges observed produced uneven breaks and loss of fragments, likely candidates for micronuclei. Tracking newly formed micronuclei had shown that errors in mitosis generate DNA breaks resulting in formation of micronuclei containing whole-chromosomes [15]. These micronuclei undergo defective and asynchronous DNA replication that extensively fragments the DNA, become distributed to daughter nuclei, and can lead to mutations and chromosomal rearrangements that persist over generations [15]. Therefore, ZDV- and NVP-induced DNA damage seen in this study may persist, could lead to aneuploidy, and could be involved in tumorigenesis.

Whereas onions and children cannot be compared directly, the reversibility of genotoxicity in drug-exposed *Allium* bulbs has some similarities to findings in children exposed perinatally to ZDV. Children exposed *in utero* to ZDV for longer than 7.5 weeks had slightly lower birth weights than children born to HIV-1-uninfected mothers, and the difference was reversible by 18 months of age [7]. Monitoring of several parameters in ZDV-exposed children between 3.2–5.6 years found no significant differences in one study [5]. However, another study reported a ten-fold increase in micronucleated reticulocyte frequencies among mother-child pairs that received prenatal ZDV, but no increases were detected in those who received prenatal antiretroviral therapy without ZDV [16]. This effect was reversible, however, and the micronucleated reticulocyte level returned to normal during the first 6 months of life [16]. Micronucleated reticulocytes occur as a result of damage to the reticulocytes when the body releases cells not fully matured into the blood stream, and the immature cells retain their nuclei. It is interesting to note that the lowest drug concentrations used in the 96 hr root growth inhibition phase of our study were equivalent to the peak plasma concentrations of these drugs in patients.

The NRTIs ZDV and 3TC are mutagenic in children exposed transplacentally and in cultured cells. The glycophorin A (GPA) somatic cell mutation assay was used to screen for mutagenesis in reticulocytes taken from mother and child pairs exposed to ZDV [9]. GPA variants arising from chromosome loss, duplication and recombination were significantly elevated in the ZDV-exposed pairs [9]. A similar study reported NRTI-induced mutagenic changes in T-cells examined for *Hprt* mutant frequencies [17]. Exposure of cells either to 100 µM ZDV and 100 µM Lamivudine (3TC) for 3 days, or to peak plasma-equivalent levels, 10 µM ZDV and 10 µM 3TC for 30 days, resulted in elevated drug-induced *Hprt* mutant frequencies in mice necropsied on days 13, 15, or 21 postpartum [17]. Future developments of the *Allium* model could be enhanced by measurement of a mutagenic end point.

Here we have shown the genotoxicity of ZDV and NVP in the *Allium cepa* genotoxicity assay. Many of the high-throughput methodologies used in research are unavailable in African research institutions, and such resource-limited settings often lack the capacity for cell culture and/or specialized *in vitro* models. The Replace-Reduce-Refine (3Rs) initiative was first articulated in 1959 to encourage scientists to reduce the number of animal models being used for research. As they reduce the number, they were further encouraged to refine the methodologies used for crucial animal research and ultimately to replace the animal models with other models. The plant model *Allium cepa* has been used extensively in research and here we demonstrated genotoxicity of ZDV and NVP consistent with previous findings using, for example, HeLa cell lines [12], [13], [14].

The *Allium* test is highly sensitive and positive toxic effects may result for compounds which may not be harmful when tested in other systems. However, false negatives have been shown to rarely occur in the *Allium* test [18], thus any compound giving a negative result can be reliably considered non-mutagenic. This test has shown good agreement with results from other test systems, even those using prokaryotic and eukaryotic organisms [10]. However, extrapolating results from one test system to another should be based on the results of a battery of tests and with due consideration to the metabolic pathways of the compound tested.
